# Loss of *Myostatin* Alters Gut Microbiota and Carbohydrate Metabolism to Influence the Gut–Muscle Axis in Cattle

**DOI:** 10.3390/vetsci12060560

**Published:** 2025-06-07

**Authors:** Chao Hai, Hongyu Gong, Yanan Xu, Xuefei Liu, Chunling Bai, Guanghua Su, Lei Yang, Guangpeng Li

**Affiliations:** State Key Laboratory of Reproductive Regulation and Breeding of Grassland Livestock, College of Life Science, Inner Mongolia University, Hohhot 010070, China; h15248037201@163.com (C.H.); vickygonghy@163.com (H.G.); 17824230613@163.com (Y.X.); liuxuefei1006@126.com (X.L.); 111980289@imu.edu.cn (C.B.); guanghuasu@imu.edu.cn (G.S.)

**Keywords:** *myostatin* gene-editing, gut microbiota, glycolysis/gluconeogenesis, *Bacteroides*

## Abstract

The gut–muscle axis plays a vital role in host metabolism and health. Although the *MSTN* gene is a well-known negative regulator of muscle growth, its role in intestinal function and metabolism remains unclear. Understanding this connection is crucial for revealing the systemic impact of *MSTN* gene editing and its potential to improve metabolic efficiency in livestock. In this study, we investigated the influence of *MSTN* deletion on gut microbiota composition and carbohydrate metabolism in the cecum and colon of cattle. Using integrated metagenomic, metabolomic, serum biochemical, and muscle transcriptomic analyses, we found significant alterations in microbial communities and key metabolic pathways. *Hallella* and *Escherichia* in the colon, as well as *Alishewanella* in the cecum, were closely linked to carbohydrate metabolism. Differential microbes and metabolites influenced key metabolic pathways, including glycolysis/gluconeogenesis and lipopolysaccharide biosynthesis. Functional gene analysis identified *Bacteroides* as the most critical bacterium affecting glycolysis/gluconeogenesis. Additionally, genes related to carbohydrate esterases were upregulated. These changes correlated with reduced serum glucose and insulin levels while increasing muscle gene expression related to glucose-to-lactose conversion. Overall, *MSTN* gene editing alters gut microbiota composition and carbohydrate metabolism in the cecum and colon, thereby influencing host glucose metabolism and energy homeostasis.

## Simple Summary

The gut–muscle axis is essential for maintaining host energy balance. In this study, we explored how editing the *myostatin* (*MSTN*) gene, which regulates muscle growth, affects the intestinal microbiota and metabolism in cattle. We found that *MSTN* gene-edited cattle exhibited distinct microbial communities in their cecum and colon, particularly involving bacteria associated with carbohydrate metabolism. These microbial changes were linked to enhanced glycolysis/gluconeogenesis activity and shifts in related enzyme genes, especially those from *Bacteroides*. The altered gut environment also influenced blood glucose and insulin levels and promoted glucose utilization in muscle. These findings suggest that *MSTN* gene editing not only affects muscle development but also reshapes intestinal metabolism, contributing to systemic metabolic regulation through the gut–muscle axis.

## 1. Introduction

The mammalian gut microbiota has always been a focal point of interest for researchers, and its interaction with the host is crucial. A stable and balanced gut microbiota plays a significant role in the physiological and metabolic activities of humans and animals [1,2]. Therefore, the gut microbiota is a key area of research for studying the unknown or unintended effects of transgenic or gene-edited animals. The dynamic balance of the gut microbiota is a hallmark of host intestinal health and is the foundation of healthy growth [3,4]. The gut, as a central site for nutrient absorption, undergoes dynamic microbial shifts that not only influence host health but also modulate the fermentation of carbohydrates and the production of key metabolites such as short-chain fatty acids (SCFAs) and branched-chain amino acids (BCAAs) [5,6,7]. These metabolites are essential for maintaining energy balance, metabolic homeostasis, and muscle function. Notably, SCFAs are produced during microbial fermentation and can either serve as fuel for colonocytes or be absorbed into the systemic circulation. In skeletal muscle, SCFAs can be oxidized directly, incorporated into glucose via gluconeogenesis, or enhance the bioavailability of glucose, glycogen, and fatty acids during physical activity [8]. These processes collectively support improved exercise performance and energy metabolism. The large intestine of mammals constitutes a vital part of the gut. While ruminants, such as cattle, possess a robust stomach structure for fermentative metabolism of feed, facilitating nutrient absorption in the small intestine, there are still nondigestible carbohydrates (NDCs) that escape absorption in the stomach and small intestine and undergo fermentation by microbes in the cecum and colon of the large intestine [9]. Despite a reduction in cellulose-degrading microbes, there is a high abundance of microbes involved in the breakdown of soluble polysaccharides derived from primary cellulose degradation [10].

*MSTN*, a key regulator of muscle growth and development, significantly impacts metabolic processes in animals. Increasing evidence suggests that *MSTN* influences whole-body metabolism, including lipid and SCFAs metabolism, blood metabolite profiles, energy homeostasis, and reproductive metabolic processes [4,11,12,13]. Our previous work also demonstrated that the rumen microbiota in *MSTN* gene-edited cattle enhances muscle growth by modulating BCAAs metabolism [14]. These findings support a broader regulatory role of *MSTN* in systemic metabolic reprogramming. However, there are few reports on whether alterations in the *MSTN* gene affect the absorption of NDCs in the large intestine.

While *MSTN* gene editing is known to enhance muscle development, its potential systemic effects, particularly on the gut microbiota and subsequent carbohydrate metabolism in the large intestine, remain largely unexplored. This study addresses this knowledge gap by characterizing microbial and metabolic changes in the cecum and colon of *MSTN* gene-edited cattle compared to wild-type (WT) controls, thereby providing new insights into gene–microbiota–metabolism interactions.

## 2. Materials and Methods

### 2.1. Animals

*MSTN* gene-edited cattle and WT cattle, all of Mongolian breed, were bred and genotyped in our laboratory [15]. This approach utilized sgRNAs designed to target exon 1 of the *MSTN* gene, followed by transfection into bovine fetal fibroblasts (BFFs) for gene editing. Successfully edited cells were screened and subsequently used as donor cells for somatic cell nuclear transfer (SCNT) to generate cloned bulls. To ensure genetic consistency, both *MSTN* gene-edited and WT cattle were derived from the same breed, with the only difference being the targeted modification in the *MSTN* gene. To further minimize environmental variability and batch effects, we selected 5 *MSTN* gene-edited male cattle and 4 age-matched WT male cattle (approximately 2 years old), and all cattle were slaughtered in the same facility and on the same day, and tissues were collected, frozen, and processed simultaneously. This approach helps eliminate time- and handler-induced variation in downstream molecular analyses. Additionally, all animals were managed by the same staff on the same farm, received the same total mixed ration (TMR) diet, and lived under uniform housing, lighting, and climate conditions, ensuring a highly controlled experimental environment. Table 1 and Table 2 provided detailed statistics on feed composition. Our experimental subjects were raised in Hohhot, China, at an average elevation of 1040 m, with a relative humidity of 48%, a mean annual temperature of 14 °C, and an average of 9.3 h of sunshine per day, providing a consistent environmental background for our investigation.

### 2.2. Cecum and Colon Fluid Sampling

Cecum and colon fluid samples were obtained from both *MSTN* gene-edited cattle and WT cattle within 2 h post-slaughter. The cecum and colon contents were manually squeezed to extract the cecum and colon fluid sample. Additionally, the cecum and colon fluid sample contained small particles. The bottle containing the cecum and colon fluid sample was promptly placed in an icebox to prevent microbial contamination. Ruminal fluid samples from each cattle were divided into triplicates, transferred to the laboratory, and stored at −80 °C until microorganism DNA extraction and cecum and colon metabolite extraction.

### 2.3. Hematoxylin–Eosin (HE) Staining

After slaughter, the cecum and colon were quickly cut into approximately 0.5 cm × 0.5 cm specimens using sterile surgical blades and washed with PBS. The cells were then fixed in 4% paraformaldehyde for 24 h at room temperature. Fixed tissues were then dehydrated through a graded ethanol series (70%, 80%, 95%, and 100%), cleared in xylene, and embedded in paraffin using an automatic tissue processor (Leica TP1020, Leica Biosystems, Wetzlar, Germany). After fixation, the samples were embedded in paraffin blocks and sectioned into 2–4 μm slices using a Leica SM 2000 R microtome. The slices were dried overnight at 37 °C.

For histological analysis, paraffin sections were deparaffinized in xylene and rehydrated through a graded ethanol series, and stained with hematoxylin for 5 min followed by eosin for 2 min, as previously described [16]. After staining, slides were dehydrated through graded alcohols, cleared in xylene, and coverslipped using mounting medium. Images were captured using a light microscope (Nikon Eclipse E100, Tokyo, Japan) equipped with a digital camera.

### 2.4. Metagenomic Sequencing and Analysis

We extracted DNA from cecum and colon fluid samples using the E.Z.N.A. Stool DNA Kit (D4015-02, Omega Bio-tek, Norcross, GA, USA) and stored it at −80 °C until PCR. DNA libraries were prepared using the TruSeq Nano DNA LT Library Preparation Kit (FC-121-4001, Illumina, San Diego, CA, USA), and metagenome sequencing was conducted using the HiSeq4000 platform (Illumina, San Diego, CA, USA).

To ensure high-quality data for subsequent analysis, we processed the raw sequencing reads as follows: first, sequencing adapters were removed using Cutadapt v1.9 with default parameters [17]. Second, low-quality reads (quality scores < 20), short reads (<100 bp), and reads containing more than 5% “N” records were trimmed in Fqtrim v0.9.4 [18] with a sliding window algorithm. Third, the reads were aligned to the host genome (Bos_taurus_UMD_3.1) using Bowtie2 v2.2.0 [19] to eliminate host contamination. After obtaining quality-filtered reads, de novo assembly was performed using IDBA-UD v1.1.1 [20] to construct the metagenome for each sample. Contigs longer than 500 bp were retained for subsequent clustering analysis. The assembly quality was evaluated using the QUAST program. MetaGeneMark v3.26 [21] was employed to predict coding sequences (CDS) from contigs ≥500 bp. CDS sequences shorter than 100 nt were filtered out based on the prediction results. The remaining CDS sequences were clustered using CD-HIT (v4.6.1) [22] with identity ≥ 95% and coverage ≥ 90% to generate representative unigenes. Clean data from each sample were aligned to the gene sequences using Bowtie2, and the number of reads mapped to each gene was counted (Supplementary Table S1). Genes with reads mapped ≤ 2 across all samples were filtered out, resulting in the final set of unigenes for further analysis. The abundance of each gene in each sample was calculated based on the number of mapped reads and the length of the gene.

Taxonomic classification of unigenes was achieved by aligning them against the NCBI NR database (v20230717) using DIAMOND v0.9.14 with the BLASTp algorithm (e-value ≤ 1 × 10^−5^). For each unigene, the alignment results with e-values ≤ 10 times the minimum e-value were selected for species classification. Species annotation was carried out using a method similar to the lowest common ancestor (LCA) algorithm in MEGAN software v6.25.10, incorporating the NCBI taxonomic classification system. This allowed us to obtain species annotation information across various taxonomic levels (SuperKingdom, Phylum, Class, Order, Family, Genus, Species). The abundance of information of unigenes was combined with species classification to derive abundance at each taxonomic level, calculated as the sum of the abundances of unigenes annotated to that taxonomic level. The Wilcox test was used to identify the differentially abundant species, and significances were declared at *p* < 0.05 and |log2-fold change| > 1. We then analyzed differences in microorganism abundance at different taxonomic levels. The GO, KEGG, CAZy (v20220806), and eggNOG (v4.5) databases were used for functional analysis.

### 2.5. Untargeted Metabolome Analysis

The cecum and colon fluid samples were thawed on ice, and 20 μL of each sample was mixed with 120 μL of precooled 50% methanol, vortexed, and subsequently incubated. After incubation, the samples were stored overnight at −20 °C. The supernatants were then transferred to fresh 96-well plates after centrifugation at 4000× *g* for 20 min. Prior to LC–MS analysis, all materials were maintained at −80 °C. LC–MS data acquisition followed the instrument’s protocols (quality control: a mixture of 10 μL per sample). Chromatographic separations were conducted using an ultra-performance liquid chromatography (UPLC) system (SCIEX, Warrington, UK) equipped with an ACQUITY UPLC T3 column (100 mm × 2.1 mm, 1.8 µm, Waters, Wilmslow, UK) for reversed-phase separation at 35 °C.

The LC–MS raw data files were first converted to mzXML format using MSConvert and then imported into the XCMS package [23] implemented in R for peak detection and retention time alignment. The CAMERA package [24] was used for isotope annotation and adduct deconvolution. Metabolite annotation was performed using the metaX toolbox [25] by matching accurate *m*/*z* values and retention times, with a mass accuracy threshold typically within ±10 ppm to the KEGG and HMDB databases. Secondary MS spectra were matched against in-house metabolite standards for further identification. To ensure data quality, features missing in more than 50% of QC samples or more than 80% of experimental samples were removed, and missing values were imputed using the K-nearest neighbor (KNN) method. Signal drift correction was carried out using QC-RLSC normalization, and features with a coefficient of variation (CV) greater than 30% in QC samples were excluded. For statistical analysis, univariate tests (Student’s *t*-test with BH correction) were conducted. Metabolites with fold change ≥ 2 and adjusted *p*-value < 0.05 were considered significantly different.

### 2.6. Serum Glucose and Insulin Measurements

Serum glucose was determined using a Cobas c311 analyser (Roche, Basel, Switzerland). The serum insulin concentration was determined by enzyme-linked immunosorbent assay (Insulin, Bovine ELISA, Mercodia, Uppsala, Sweden). A 25 μL serum sample was obtained from *MSTN* gene-edited and WT cattle. The samples were enzyme-conjugated and incubated on a plate shaker at 800 rpm for 2 h at room temperature (18–25 °C). After incubation, the samples were washed 6 times with 700 µL of wash buffer (1× solution) per tube. Subsequently, 200 μL of Substrate TMB was added to each well and incubated for 15 min at room temperature (18–25 °C). Finally, 50 μL of Stop Solution was added to each well. The optical density was read at 450 nm, and the results were calculated accordingly.

### 2.7. Transcriptome Analysis

The total RNA was isolated using TRIzol reagent (Invitrogen, Carlsbad, CA, USA), and RNA integrity was assessed using an Agilent Bioanalyzer 2100, with samples exhibiting RIN values > 7.0. Poly(A) mRNA was purified using Dynabeads Oligo(dT)25, fragmented, and reverse transcribed to cDNA using SuperScript II Reverse Transcriptase. Second-strand cDNA synthesis, end repair, A-tailing, adaptor ligation, and size selection (average insert size 300 ± 50 bp) were performed according to standard protocols. The libraries were PCR-amplified and subjected to paired-end sequencing (2 × 150 bp) using the Illumina NovaSeq 6000 platform.

The raw sequencing data were subjected to quality control, including the removal of reads containing adaptor contamination using Cutadapt software (v1.9) with parameters set as: minimum overlap = 5 bp, and minimum read length = 100 bp. After removing low-quality and undetermined bases, we used HISAT2 software (v2-2.0.4) [26] to map the reads to the genome (Bos_taurus_UMD_3.1) using paired-end reads. The mapped reads of each sample were assembled using StringTie (v1.3.4d) [27] with default parameters. Then, all the transcriptomes from all the samples were merged to reconstruct a comprehensive transcriptome using gffcompare software (v0.12.6). After the final transcriptome was generated, StringTie and Ballgown were used to estimate the expression levels of all the transcripts, and the expression levels of the mRNAs were determined by calculating the FPKM.

To identify differentially expressed mRNAs, the R package DESeq2 [28] was used to select transcripts with a fold change >2 or <0.5 and a *p* value < 0.05. Gene ontology (GO) enrichment and KEGG enrichment analyses (Benjamini–Hochberg-corrected *p*-value < 0.05) were performed on the differentially expressed mRNAs using specialized tools [29,30].

### 2.8. Data Analysis

Statistical analysis and mapping of glucose and insulin measurement data were performed using GraphPad Prism v8.3.0. Principal component analysis (PCA), volcano plot analysis, and α diversity analysis were performed with R v4.2.1. The level of significance was indicated by * *p* < 0.05, ** *p* < 0.01, *** *p* < 0.001.

## 3. Results

### 3.1. Characterization of Gut Microbiota and Histological Features in Cecum and Colon

We performed metagenomic analysis of the cecum and colon contents from *MSTN* gene-edited and WT cattle, revealing differences in 1959 microbial genera involved in non-digestible carbohydrate metabolism.

**Figure 1:**
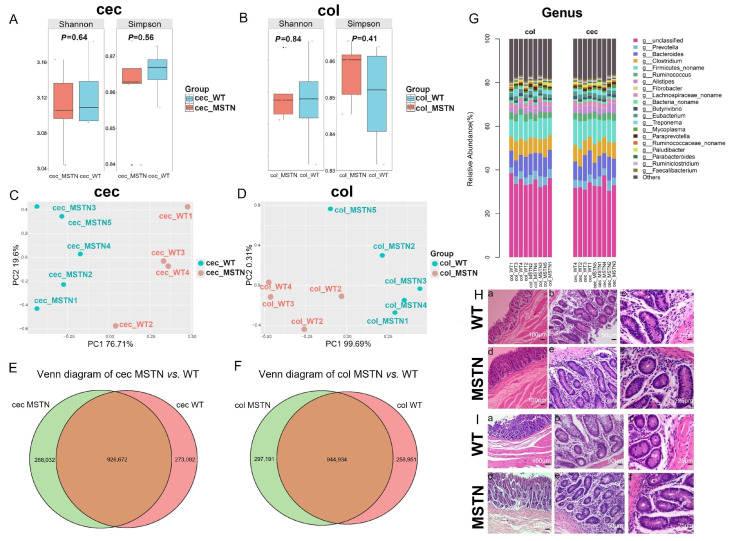
Distribution, diversity of microorganisms, and histological features in the cecum and colon of *MSTN* gene-edited cattle and WT cattle. (**A**) Analysis of the α diversity indices of cecal (cec) microorganisms. (**B**) Analysis of the α diversity indices of colonic (col) microorganisms. (**C**) PCA of cecal microorganisms. (**D**) PCA of colonic microorganisms. (**E**) Volcano plot analysis of cecal microorganism unigenes. (**F**) Volcano plot analysis of colonic microorganism unigenes. (**G**) Analysis of the relative abundance of cecal and colonic microbial genera. (**H**) Analysis of caecal wall and villus morphology. (**a**–**c**): WT cattle, (**d**–**f**): *MSTN* gene-edited cattle. (**I**) Analysis of the morphology of the colonic wall and villus morphology. (**a**–**c**): WT cattle, (**d**–**f**): *MSTN* gene-edited cattle (μm).

Although α-diversity indices (Shannon and Simpson) did not show significant differences between the two groups (Figure 1A,B), PCA analysis revealed significant differences in the microbial community composition of the cecum and colon between *MSTN* gene-edited and WT cattle (Figure 1C,D).

We next compared the unigenes identified in both tissues. In the cecum, *MSTN* gene-edited and WT cattle shared 926,672 unigenes, while 288,032 unigenes were unique to the *MSTN* group and 273,082 were unique to WT cattle. In the colon, 944,934 unigenes were shared, with 279,191 unigenes unique to *MSTN* gene-edited cattle and 258,951 unique to WT cattle (Figure 1E,F).

Further taxonomic analysis showed that the predominant microbial genera in both groups included *Prevotella*, *Bacteroides*, *Clostridium*, *Firmicutes_noname*, *Ruminococcus*, and *Alistipes.* However, no significant differences were observed in the relative abundances of these high-abundance genera between *MSTN* gene-edited and WT cattle (Figure 1G).

Histological examination revealed no pathological alterations in the intestinal structure of *MSTN* gene-edited cattle. In the cecum, the wall was clearly stratified into the mucosa, submucosa, muscularis, and serosa layers, though lacking folds or villi. The intestinal glands appeared well-developed, long, and straight. At higher magnification, numerous absorptive cells and goblet cells were observed, with no abnormal tissue architecture (Figure 1H). Similarly, the colon exhibited no circular folds or villi, but the intestinal glands were clearly developed, and many goblet cells were present at high magnification. No pathological changes or structural abnormalities were observed in the MSTN gene-edited group compared to WT cattle, indicating that gene editing did not adversely affect colon histology (Figure 1I).

### 3.2. Differential Microorganisms in the Cecum and Colon Influence the Glycolysis/Gluconeogenesis Pathway

**Figure 2:**
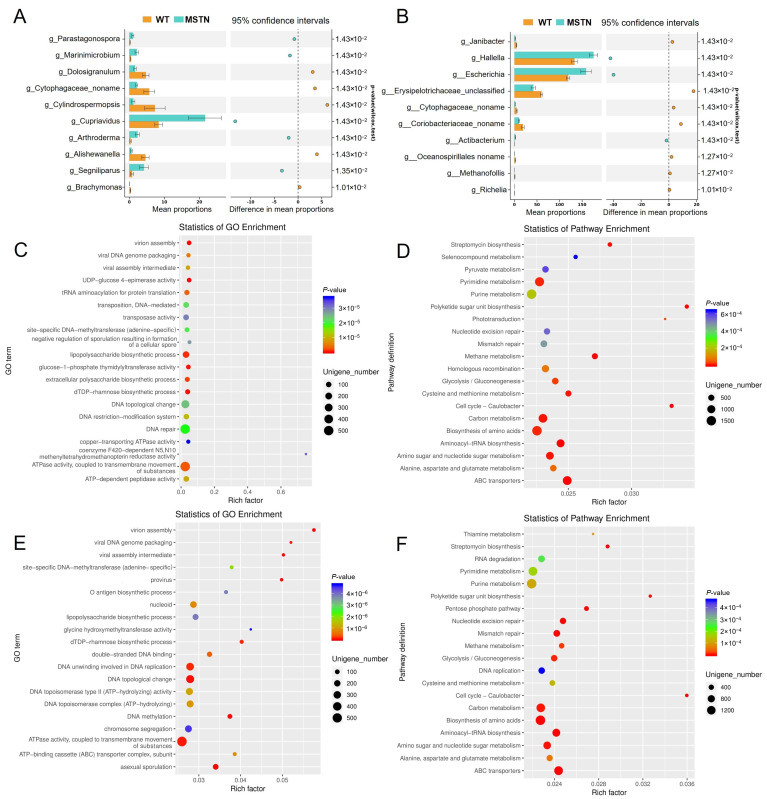
GO and KEGG functional analyses of the differentially abundant microorganisms. (**A**) Stamp analysis of the top ten differential microorganisms in the cecum. (**B**) Stamp analysis of the top ten differential microorganisms in the colon. (**C**) GO analyses of differentially abundant microorganisms in the cecum. (**D**) KEGG analyses of differentially abundant microorganisms in the cecum. (**E**) GO analyses of differentially abundant microorganisms in the colon. (**F**) KEGG analyses of differentially abundant microorganisms in the colon.

The above results indicate that the morphology and abundance of microorganisms in the cecum and colon did not significantly change, but the distribution of microorganisms differed. Stamp differential analysis identified the top ten genera with significantly different abundances in the cecum, which included *Parastagonospora*, *Marinimicrobium*, *Dolosigranulum*, *Cytophagaceae_noname*, *Cylindrospermopsis*, *Cupriavidus*, *Arthroderma*, *Alishewanella*, *Segniliparus*, and *Brachymonas* (Figure 2A, Supplementary Table S2). In the colon, the top ten significantly different genera were *Janibacter*, *Hallella*, *Escherichia*, *Erysipelotrichaceae_unclassified*, *Cytophagaceae_noname*, *Coriobacteriaceae_noname*, *Actibacterium*, *Oceanospirillales_noname*, *Methanofollis*, and *Richelia* (Figure 2B, Supplementary Table S3).

To explore the potential metabolic impacts of these changes, we performed functional enrichment analyses. GO analysis revealed enrichment in carbohydrate metabolism-related processes, including UDP-glucose 4-epimerase activity, lipopolysaccharide biosynthetic process, glucose-1-phosphate thymidylyltransferase activity, and extracellular polysaccharide biosynthetic process (Figure 2C). Consistent with this, KEGG pathways analysis identified alterations in glycolysis/gluconeogenesis, pyruvate metabolism, biosynthesis of amino acids, amino sugar and nucleotide sugar metabolism, and ABC transporters (Figure 2D).

In the colon, GO enrichment indicated involvement in the lipopolysaccharide biosynthetic process and ATPase activity (Figure 2E). KEGG analysis of colon microbiota echoed the cecal findings, highlighting shifts in ABC transporters, glycolysis/gluconeogenesis, and methane metabolism (Figure 2F). Together, these results suggest that *MSTN* gene editing similarly affects the cecum and colon, primarily by influencing carbohydrate metabolism.

### 3.3. Bacteroides Is the Key Microbe in the Glycolysis/Gluconeogenesis Pathway

**Figure 3:**
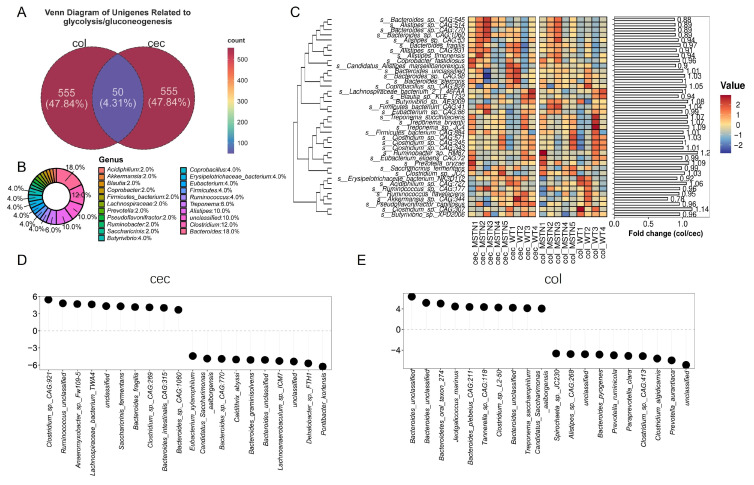
Analysis of microorganisms related to glycolysis/gluconeogenesis in the cecum and colon. (**A**) Venn diagram of unigenes related to glycolysis/gluconeogenesis. (**B**) Microbial genera corresponding to the 50 common differential unigenes. (**C**) Abundance and distribution of species involved in glycolysis/gluconeogenesis in the cecum and colon. (**D**) Top 10 microbial taxa with increased and decreased involvement in the glycolysis/gluconeogenesis pathway in the cecum of *MSTN* gene-edited cattle. (**D**,**E**) Top 10 microbial taxa with increased and decreased involvement in the glycolysis/gluconeogenesis pathway in the colon of *MSTN* gene-edited cattle.

Next, we investigated microorganisms associated with the glycolysis/gluconeogenesis pathways. In total, we identified 605 differentially abundant unigenes related to glycolysis/gluconeogenesis across the cecum and colon, with 50 unigenes showing common differences (Figure 3A). Taxonomic analysis revealed that these shared unigenes were predominantly affiliated with the genus *Bacteroides* (18%), *Clostridium* (12%, Figure 3B). These unigenes belong to 39 species, which displayed similar abundance patterns in both the cecum and colon. Notably, most *Bacteroides* species were less abundant in the cecum compared to the colon. For instance, *Bacteroides* sp. *CAG:545* (col/cec fold change = 0.88), *Bacteroides* sp. *CAG:770* (col/cec fold change = 0.89), and *Bacteroides* sp. *CAG:1060* (col/cec fold change = 0.89). In contrast, species from the genus *Clostridium* were more abundant in the colon, such as *Clostridium* sp. *CAG:571* (col/cec fold change = 1.03), *Clostridium* sp. *CAG:343* (col/cec fold change = 1.01), *Clostridium* sp. *JCC* (col/cec fold change = 1.03), and *Clostridium* sp. *CAG:307* (col/cec fold change = 1.14) (Figure 3C).

In the cecum, there was an increase in *Clostridium* sp. *CAG:921 Bacteroides fragilis*, *Bacteroides* sp. *CAG:1060*, and a decrease in the abundance of *Bacteroides intestinalis CAG:770* (Figure 3D). In the colon, *MSTN* gene editing led to increased levels of *Bacteroides oral taxon 274*, *Bacteroides plebeius CAG:211*, and other unclassified *Bacteroides* species, while the abundance of *Bacteroides pyogenes*, *Clostridium* sp. *CAG:413*, and *Clostridium algidicamis* decreased (Figure 3E).

Together, our results demonstrate that *MSTN* gene editing significantly altered the microbiota of the *Bacteroides* genera, leading to changes in glycolysis/gluconeogenesis metabolism.

### 3.4. Metabolomic Profiling and Carbohydrate Metabolism Pathways

**Figure 4:**
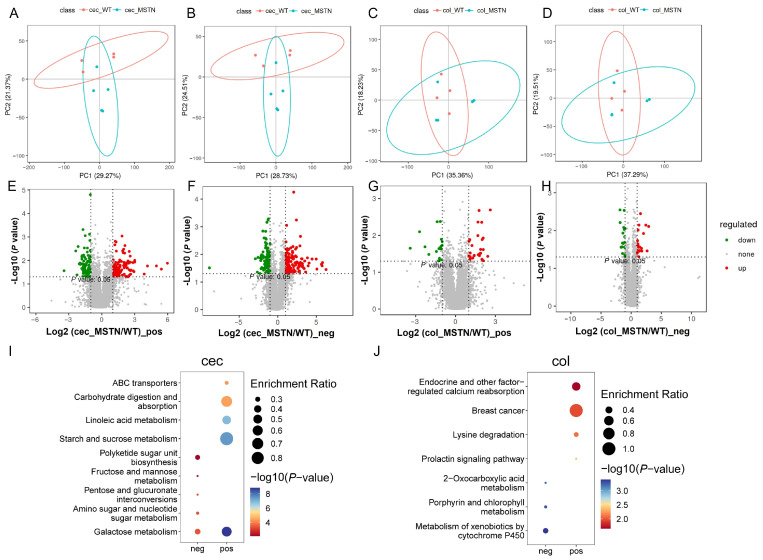
Functional analysis of differentially abundant metabolites. (**A**) PCA of metabolites in the cecum in the pos modes. (**B**) PCA of metabolites in the cecum in the neg modes. (**C**) PCA of metabolites in the colon in the pos modes. (**D**) PCA of metabolites in the colon in the neg modes. (**E**) Volcano plot of differentially abundant metabolites in the pos modes in the cecum. (**F**) Volcano plot of differentially abundant metabolites in the neg modes in the cecum. (**G**) Volcano plot of differentially abundant metabolites in the colon in the pos groups. (**H**) Volcano plot of differentially abundant metabolites in the colon in the neg groups. (**I**) Pathway analysis of differentially abundant metabolites in the cecum. (**J**) Pathway analysis of differentially abundant metabolites in the colon.

Gut metabolites play a critical role in maintaining intestinal homeostasis. To explore whether the observed microbial shifts influenced the metabolic landscape, we next examined the metabolomic profiles of the cecum and colon. The cecum exhibited more pronounced metabolic alterations compared to the colon (Figure 4A–D). In the cecum, we identified 214 significantly altered metabolites in the pos ion mode (120 upregulated, 94 downregulated) and 229 in the neg ion mode (132 upregulated, 97 downregulated). By contrast, the colon showed fewer changes, with only 45 and 41 significantly different metabolites detected in the positive and negative ion modes, respectively (Figure 4E–H).

Functional enrichment analysis revealed that these metabolic changes were highly consistent with our metagenomic findings. In the cecum, altered metabolites were predominantly enriched in carbohydrate metabolism pathways, including galactose metabolism, amino sugar and nucleotide sugar metabolism, and starch and sucrose metabolism, indicating that *MSTN* gene editing altered carbohydrate metabolism in the cecum (Figure 4I, Supplementary Table S4). In contrast, the colon showed enrichment in distinct metabolic pathways, such as lysine degradation, metabolism of xenobiotics by cytochrome P450, and the prolactin signaling pathway (Figure 4J, Supplementary Table S5). These findings indicate that while *MSTN* gene editing affects both gut regions, the cecal metabolic response is more pronounced and more tightly linked to microbial carbohydrate processing.

### 3.5. Key Differential Microbes Exhibit Metabolic Links to Carbohydrate Metabolism

**Figure 5:**
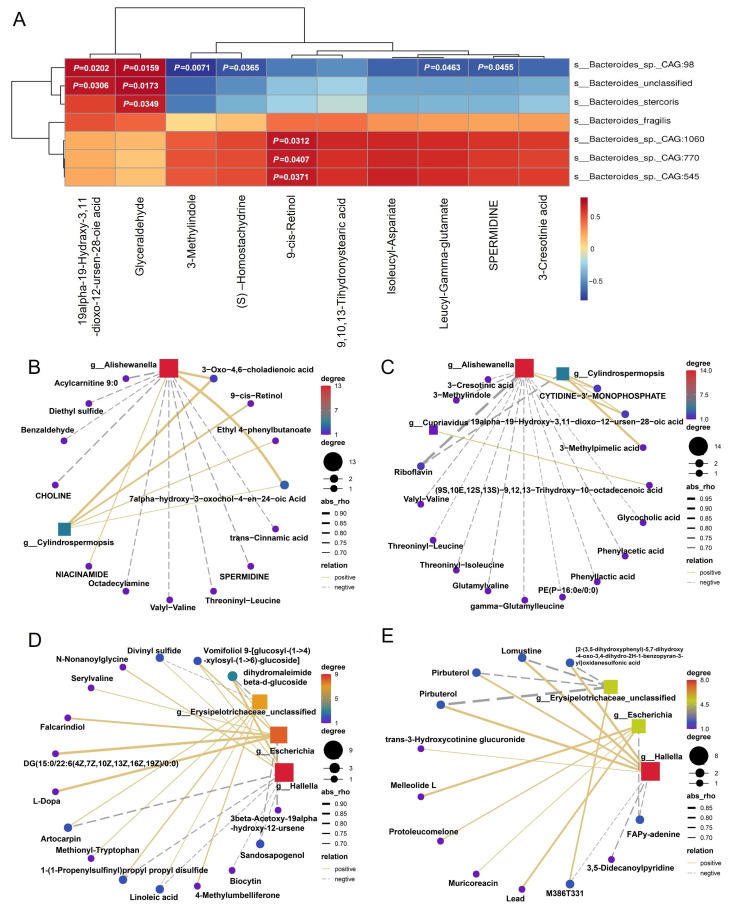
Correlation network of metabolites and microbial correlations. (**A**) Associations between *Bacteroides* species related to glycolysis/gluconeogenesis and corresponding metabolites identified in both the cecum and colon. (**B**) Correlation network analysis of differential microorganisms and metabolites in the pos mode in the cecum. (**C**) Correlation network analysis of differential microorganisms and metabolites in the neg mode in the cecum. (**D**) Correlation network analysis of differentially abundant microorganisms and metabolites in the colon in pos mode. (**E**) Correlation network analysis of differentially abundant microorganisms and metabolites in the neg mode in the colon.

Based on previous findings, several key *Bacteroides* species, such as *Bacteroides fragilis*, *Bacteroides* sp. *CAG:770*, *CAG:1060*, and *Bacteroides intestinalis* were identified as important contributors to the glycolysis/gluconeogenesis pathway. Correlation analysis between these species and metabolites (Top 10 correlations) revealed a positive association with *Glyceraldehyde*, a central glycolytic intermediate. Specifically, *Bacteroides_*sp.*_CAG:98* (*p* = 0.0159), *Bacteroides_unclassified* (*p* = 0.0173), and *Bacteroides_stercoris* (*p* = 0.0349) (Figure 5A). This suggests that *MSTN* gene editing may modulate glucose metabolism by influencing the abundance and metabolic activity of glycolysis-related *Bacteroides*.

To further investigate the involvement of significantly altered microorganisms that are also involved in carbohydrate metabolism, we selected the three most abundant differential genera from the cecum and colon for correlation analysis with differential metabolites. In the cecum, *Alishewanella* had a strong positive correlation with 3-oxo-4,6-choladienoic acid and 7 alpha-hydroxy-3-oxochol-4-en-24-oic acid in pos ion mode. Similarly, *Cylindrospermopsis* also had a strong positive correlation with 3-oxo-4,6-choladienoic acid, indicating that the high abundance of these two bacteria in WT cattle may be related to 3-oxo-4,6-choladienoic acid (28,130.23 ± 18,315.29 vs. 97,946.14 ± 34,423.66, *p* = 0.0056, Supplementary Table S6). However, the high abundance of *Cupriavidus* in the *MSTN* gene-edited group did not significantly correlate with metabolites in the pos mode (Figure 5B).

In neg ion mode, *Cupriavidus* displayed a weak correlation with (9S,10E,12S,13S)-9,12,13-trihydroxy-10-octadecenoic acid, suggesting a more complex or indirect metabolic association. Meanwhile, both *Alishewanella* and *Cylindrospermopsis* were positively correlated with 19alpha-19-hydroxy-3,11-dioxo-12-uren-28-oic acid and negatively correlated with riboflavin (Figure 5C).

In the colon, *Hallella* and *Escherichia*, enriched in the *MSTN* gene-edited cattle, were positively correlated with the metabolites dihydromaleimide beta-D-glucoside and vomifoliol 9-[glucosyl-(1->4) xylosyl-(1->6)-glucoside] in pos ion mode. Conversely, *Erysipelotrichaceae_unclassified*, more abundant in WT cattle, was negatively correlated with dihydromaleimide beta-D-glucoside. Dihydromaleimide beta-D-glucoside emerged as a key metabolite associated with all three differential microorganisms (Figure 5D, Supplementary Table S7).

In neg ion mode, *Hallella* showed positive correlated with narirutin 4′-glucoside, pirbuterol, and lomustine, whereas *Erysipelotrichaceae_unclassified* showed negative correlations with the same metabolites (Figure 5E). These findings suggest that *MSTN* gene editing reshapes gut microbial composition in ways that impact carbohydrate and xenobiotic metabolism, reinforcing the tight coupling between microbial shifts and metabolic outputs in both the cecum and colon.

### 3.6. Integration of Transcriptomic Data with Systemic Metabolism

**Figure 6:**
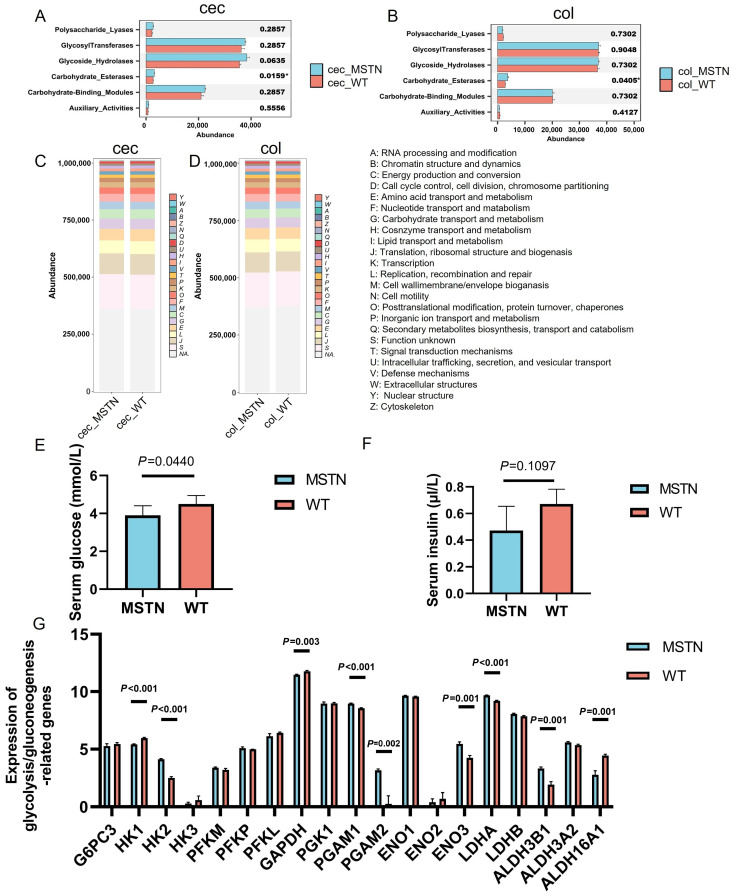
Carbohydrase and related counterpart metabolic analyses. (**A**) Cecum carbohydrase analysis. (**B**) Colonic carbohydrase analysis. (**C**) Cecum carbohydrase analysis. (**D**) Colonic carbohydrase analysis. (**E**) Comparison of serum glucose levels (mmol/L). (**F**) Comparison of serum insulin levels (μL/mL). (**G**) Expression of genes (FPKM) related to glycolysis/gluconeogenesis pathway in muscle tissue. Data are presented as mean ± standard deviation (SD). * indicates a statistically significant difference (*p* < 0.05).

The above results suggest that *MSTN* gene editing in cattle alters the microbiota in the cecum and colon, impacting carbohydrate metabolism. Carbohydrate-active enzymes, such as glycoside hydrolases, polysaccharide lyases, glycosyltransferases, and carbohydrate esterases, are key mediators of microbial carbohydrate degradation and utilization [31]. We therefore analyzed the abundance of genes encoding these enzymes in the cecum and colon. Notably, carbohydrate esterases were significantly increased in both the cecum (*p* = 0.0159) and colon (*p* = 0.0405), while no significant changes were observed for other enzymes (Figure 6A,B, Supplementary Tables S8 and S9).

Further functional annotation using the eggNOG database revealed a trend toward increased microbial capacity for carbohydrate transport and metabolism in *MSTN* gene-edited cattle, although these changes did not reach statistical significance (Figure 6C,D). This microbial remodeling may facilitate greater production of energy-yielding metabolites such as glucose, acetate, and propionate, potentially contributing to systemic energy balance.

To evaluate this possibility, we measured serum glucose and insulin levels in *MSTN* gene-edited cattle and WT cattle. *MSTN* gene-edited cattle exhibited significantly lower serum glucose levels compared to WT controls (*p* = 0.044), along with a non-significant downward trend in insulin levels (*p* = 0.1097, Figure 6E,F). These results suggest enhanced glucose utilization and improved metabolic efficiency in *MSTN*-edited animals.

At the host tissue level, transcriptomic analysis of muscle tissue revealed upregulation of key genes in the glycolysis/gluconeogenesis pathway, particularly those catalyzing the conversion from 3-phosphoglycerate to lactate (*PGAM1*, *p* < 0.001; *PGAM2*, *p* = 0.002; *ENO3*, *p* = 0.001; LDHA, *p* < 0.001) (Figure 6G). This gene expression pattern indicates an enhanced capacity for anaerobic glucose metabolism in muscle, which may support increased muscle energy turnover and growth.

Together, these findings support a mechanistic model in which *MSTN* gene editing reshapes gut microbial communities to favor enhanced carbohydrate breakdown and metabolite availability. These changes, in turn, influence systemic glucose metabolism and synergize with upregulated glycolytic activity in muscle tissue, thereby contributing to improved energy supply and muscle development in *MSTN* gene-edited cattle.

## 4. Discussion

The mutualistic relationship between symbiotic microorganisms and the host is a delicate balance maintained by proper host barrier functions and specific microbial adaptations [32]. The host genotype and gut microbiota also have an interdependent relationship. Our findings support the notion that *MSTN* gene editing in cattle alters the functional potential of the hindgut microbiota in a way that may enhance host physiological functions. Importantly, this shift occurs without significantly affecting the overall microbial composition, which is consistent with previous findings in *MSTN*-KO Meishan pigs, where no significant changes in fecal microbiota structure were observed [33]. However, a key difference lies in the microbial function: in pigs, *MSTN* deletion led to an enrichment of butyrate-producing bacteria, which was proposed to promote type IIb muscle fiber growth via activation of the Akt/mTOR pathway through G protein-coupled receptors [4]. Similarly, previous research on *MSTN* gene-edited sheep has also revealed functional remodeling of the gut microbiome. In that case, the enhancement occurred primarily through the upregulation of carotenoid biosynthesis pathways, which may improve metabolic efficiency and contribute to increased muscle mass [34]. These findings suggest that while the specific microbial mechanisms influenced by *MSTN* gene editing may differ across species, such interventions consistently appear to promote muscle development by modulating gut microbial functions in a species-specific manner.

Intestinal epithelial cells (IECs) provide physical and biochemical barriers to maintain homeostasis between host tissues and symbiotic bacteria [35]. Although significant changes in the symbiotic microbiota do not usually lead to disease, this also depends on the environment: when the mucosal surface is damaged, symbiotic microorganisms can become opportunistic pathogens and cause pathology [36]. The structure of the cecum and colon of the *MSTN* gene-edited cattle did not change, indicating that symbiotic gut microorganisms do not have the opportunity to become pathogenic to the host (Figure 1H,I).

NDCs are fermented by gut microbiota in the cecum and colon after escaping digestion in the upper gastrointestinal tract [37]. *MSTN* gene-edited cattle exhibited significant alterations in cecal and colonic microbial composition, including increased abundance of genera such as *Lactobacillus* and *Clostridium*, which are known to degrade resistant starch and non-starch polysaccharides [37,38]. These changes likely modulate carbohydrate fermentation patterns, thereby influencing host energy metabolism. Our data further showed enrichment of microbial genes involved in glycolysis/gluconeogenesis and lactose metabolism pathways, such as GALE [39,40], in *MSTN*-edited cattle. These findings suggest that *MSTN* deficiency may indirectly affect muscle energy metabolism by reshaping microbial communities and their carbohydrate metabolic functions in the hindgut.

*Bacteroides* is a major group of anaerobic bacteria widely present in the gastrointestinal tract of humans and animals, playing a crucial role in carbohydrate metabolism. These microbes can utilize various complex polysaccharides, including dietary fibers, resistant starches, and host-derived glycoproteins, as substrates [41,42]. Through their rich repertoire of carbohydrate-active enzymes, *Bacteroides* can hydrolyze these compounds into absorbable monosaccharides, which then enter glycolysis or gluconeogenesis pathways. Studies have shown that *Bacteroides* possesses a complete set of genes for the glycolytic and gluconeogenic pathways [43], and the metabolic products from these processes serve not only the bacteria themselves but also influence host metabolism by producing SCFAs such as propionate and butyrate [44]. In the host gut, this carbohydrate metabolism capacity helps shape the local energy environment, potentially enhancing glucose homeostasis, energy absorption, and even affecting skeletal muscle metabolic activity [45].

*Alishewanella* can utilize various organic and inorganic compounds as carbon and energy sources. In the blood of type 2 diabetes patients, the abundance of this genus of bacteria significantly increases, indicating its influence on the glucose metabolism process [46]. In ileal transposition (IT) surgery on diabetic Goto-Kakizaki (GK) rats, it was found that serum glucose levels significantly decreased, the content of 3-oxo-4,6-choladienoic acid increased, and the metabolism of unsaturated fatty acids, such as linolenic acid metabolism, changed. Interestingly, this study also revealed that changes in *Alishewanella* led to alterations in linoleic acid metabolism (Figure 4I). However, in this case, 3-oxo-4,6-choladienoic acid was significantly positively correlated with *Alishewanella*. Previous findings indicated that *MSTN* gene-edited cattle have lower blood glucose levels [47], and in *MSTN* gene-edited cattle, *Alishewanella* bacteria were reduced (Figure 2A and Figure 5A).

*Hallella* species are obligate anaerobic bacteria that are Gram-negative, rod-shaped, and nonspore-forming. These bacteria typically inhabit the ecological niche of anaerobic mammals and are key degraders of carbohydrates in the intestine [48]. The glycoside hydrolase (GH) family is the largest CAZyme family in this strain, followed by the glycosyl transferase (GT) family, indicating the strain’s ability to utilize polysaccharides [49]. This indicates that in this study, the higher levels of GH and GT in *MSTN* gene-edited cattle were closely related to *Hallella* (Figure 2B). The positively correlated dihydromaleimide beta-D-glucoside, vomifoliol 9-[glucosyl-(1->4) xylosyl-(1->6)-glucoside], and narirutin 4′-glucoside are likely key glycoside compounds influencing this process (Figure 5C,D).

Additionally, we found that the differentially abundant microorganisms were associated with metabolites possessing antioxidant, anti-inflammatory, and antimicrobial activities (Figure 5). Falcarindiol, a natural product belonging to a class of compounds known as polyacetylenes, is a bioactive secondary metabolite with pharmacological activities in the colon, including anti-inflammatory, antimicrobial, and anticancer properties [50]. Artocarpin, a flavonoid compound, has been shown to exhibit various biological activities, such as antioxidant, antibacterial, anti-inflammatory, and antitumor effects [51]. Muricoreacin, a sesquiterpene compound derived from plants of the genus Muricidae, has multiple bioactivities, including anticancer, anti-inflammatory, and antioxidant properties [52]. FAPy-adenine, a DNA damage product, is a base lesion repair product associated with adenine bases and typically forms when DNA is exposed to oxidizing agents or other chemicals [53]. The significant correlation of these metabolites may suggest an enrichment of DNA-related repair and protective functions (Figure 2C,E).

We acknowledge that the relatively small sample size is a limitation, due to the technical and resource challenges of producing *MSTN* gene-edited cattle via SCNT. However, strict control of breed, age, sex, and environmental conditions helped reduce individual variability and improve data reliability. Future studies with larger, more balanced cohorts are needed to validate these findings. Interventional approaches, such as microbiota transplantation or dietary modulation, could further clarify the causal roles of gut microbiota and metabolites in muscle metabolism.

## 5. Conclusions

*MSTN* gene deficiency significantly altered the gut microbiota and metabolite profiles in the cecum and colon of cattle. Increased abundance of *Bacteroides* and carbohydrate-active enzymes was linked to enhanced glycolysis/gluconeogenesis and carbohydrate metabolism. These changes likely support improved muscle development in *MSTN* gene-edited cattle. This study reveals a key connection between host gene editing and gut microbial function, offering new insights for improving livestock growth through microbiota modulation.

## Figures and Tables

**Table 1:** Feed composition.

Feed Composition	Quality (kg)/Day
Silage (kg/head)	12
Gluten (kg/head)	2
Hay (bale/head)	2
Refined feed	2.5

**Table 2:** Nutrient composition of refined feed.

Nutrients	Composition
Crude protein, not less than	16.0
Crude fat, not more than	12.0
Crude fiber, not more than	9.0
Calcium	0.5–1.8
Total phosphorus, not less than	0.4
Sodium chloride	0.8–1.5
Lysine, not less than	0.4

## Data Availability

The original contributions presented in this study are included in the article/Supplementary Material. Further inquiries can be directed to the corresponding author(s).

## Supplementary Materials

The following supporting information can be downloaded at: https://www.mdpi.com/article/10.3390/vetsci12060560/s1, Table S1: Macrogenomic sequencing data of the cecum and colon; Table S2: Differentially abundant bacterial genera in cecum (Top 10, mean ± SD); Table S3: Differentially abundant bacterial genera in colon (Top 10, mean ± SD); Table S4: Metabolic pathways of cecum differential metabolites; Table S5: Metabolic pathways of colonic differential metabolites; Table S6: Differentially abundant metabolites in cecum (Top 10, mean ± SD); Table S7: Differentially abundant metabolites in colon (Top 10, mean ± SD); Table S8: Comparison of CAZy-associated microorganisms in the MSTN and WT groups of cec; Table S9: Comparison of CAZy-associated microorganisms in the MSTN and WT groups of col.
