# Supplementary material for: Loss of Myostatin Alters Gut Microbiota and Carbohydrate Metabolism to Influence the Gut–Muscle Axis in Cattle
Source: Vet Sci. 2025 Jun 7;12(6):560. doi: 10.3390/vetsci12060560 (PMC12197488; doi:10.3390/vetsci12060560)
Supplement: Supplementary file 1 [file vetsci-12-00560-s001.zip › Animal Ethics Approval Document.pdf]

[2023] 061

我校生命科学学院教师杨磊课题组成员关于“通过改变盲肠和结肠中的微生物群来增强糖代谢，影响 MSTN 基因编辑牛的糖酵解/葡萄糖生成途径”研究经内蒙古大学生物伦理委员会审核，符合伦理原则，批准文号：IMU-CATTLE-2023-061。

The research on " *Enhance sugar metabolism by altering the microbiota, influencing the glycolysis/gluconeogenesis pathways in the cecum and colon of MSTN gene-edited cattle* " conducted by the team members of the Lei Yang lab from the College of Life Sciences has been reviewed by the Bioethics Committee of Inner Mongolia University and is in compliance with ethical principles. Approval number: IMU-CATTLE-2023-061.

生物伦理委员会主任：胡薇

Chairman of Bioethics Committee

内蒙古大学生物伦理委员会

Bioethics Committee of Inner Mongolia University

内蒙古大学科学技术处（代章）

Inner Mongolia University Division of Science and Technology

2023 年 2 月 25 日
