# Supplementary material for: Loss of Myostatin Alters Gut Microbiota and Carbohydrate Metabolism to Influence the Gut–Muscle Axis in Cattle
Source: Vet Sci. 2025 Jun 7;12(6):560. doi: 10.3390/vetsci12060560 (PMC12197488; doi:10.3390/vetsci12060560)
Supplement: Supplementary file 1 [file vetsci-12-00560-s001.zip › supplement file_R1.pdf]

Supplementary Table S1 Macrogenomic sequencing data of the cecum and colon.

Sample	Raw Data (reads)	Clean Data	Clean %	Q20 %	Q30 %	GC%	hostgenome rate%
Cec_MSTN1	93148606/13.97G	89589012/12.50G	96.18	98.17	94.78	42.97	47.10%
Cec_MSTN2	51226192/7.68G	50028594/7.12G	97.66	97.24	92.95	44.13	15.36%
Cec_MSTN3	47010032/7.05G	44790784/6.28G	95.28	97.15	92.76	43.45	0.59%
Cec_MSTN4	72599140/10.89G	70272292/9.86G	96.79	97.68	93.75	42.99	33.58%
Cec_MSTN5	41911752/6.29G	40811178/5.80G	97.37	97.13	92.72	42.86	11.42%
Cec_WT1	58773550/8.82G	56340742/7.88G	95.86	97.17	92.82	42.66	16.05%
Cec_WT2	86514514/12.98G	83009880/11.64G	95.95	98.09	94.61	43.64	40.85%
Cec_WT3	46417218/6.96G	45007172/6.34G	96.96	97.08	92.62	43.14	2.68%
Cec_WT4	51975966/7.80G	50329250/7.11G	96.83	96.83	92.09	43.15	5.02%
Col_MSTN1	52055634/7.81G	50994254/7.28G	97.96	97.22	92.89	41.70	1.34%
Col_MSTN2	59108070/8.87G	57969094/8.35G	98.07	97.04	92.57	42.91	6.72%
Col_MSTN3	53467214/8.02G	52300438/7.50G	97.82	97.04	92.64	43.61	1.12%
Col_MSTN4	81734676/12.26G	79391012/11.24G	97.13	97.62	93.64	43.00	45.61%
Col_MSTN5	50834454/7.63G	49854732/7.12G	98.07	97.33	93.15	42.20	15.85%
Col_WT1	75977394/11.40G	74178616/10.57G	97.63	97.59	93.64	43.14	35.80%
Col_WT2	107909590/16.19G	103617250/14.60G	96.02	97.73	93.64	43.21	50.49%
Col_WT3	41611794/6.24G	40952244/5.91G	98.41	97.19	92.81	41.48	2.43%
Col_WT4	119189298/17.88G	116538570/16.67G	97.78	97.91	94.19	42.80	55.17%

Supplementary Table S2 Differentially abundant bacterial genera in cecum (Top 10, mean±SD)

Genus	Mean mstn	Mean wt	*P*-value
Janibacter	1.94±1.64	4.48±0.95	0.0143
Hallella	175.21±20.09	133.07±12.39	0.0143
Escherichia	158.87±25.98	118.97±7.69	0.0143
Erysipelotrichaceae_unclassified	41.98±11.09	59.76±4.77	0.0143
Cytophagaceae_noname	1.52±0.71	4.94±2.46	0.0143
Coriobacteriaceae_noname	10.18±3.33	18.86±6.04	0.0143
Actibacterium	2.04±1.55	0.32±0.14	0.0143
Oceanospirillales_noname	0.15±0.22	2.08±1.99	0.0127
Methanofollis	0.06±0.08	0.8±0.96	0.0127
Richelia	0.06±0.03	0.46±0.39	0.0102

Supplementary Table S3 Differentially abundant bacterial genera in colon (Top 10, mean±SD)

Genus	Mean mstn	Mean wt	*P*-value

Brachymonas	1±0.66	0.22±0.12	0.0143
Segniliparus	2.14±1.14	0.43±0.08	0.0143
Alishewanella	1.63±0.82	4.72±1.8	0.0143
Arthroderma	2.13±0.68	5.69±3.08	0.0143
Cupriavidus	1.13±1.02	7.3±5.88	0.0143
Cylindrospermopsis	21.64±10.45	8.38±2.22	0.0143
Cytophagaceae_noname	2.35±1.2	0.36±0.45	0.0143
Dolosigranulum	0.62±0.62	4.61±2.08	0.0143
Marinimicrobium	4.19±2.75	0.78±0.9	0.0135
Parastagonospora	0.04±0.02	0.35±0.13	0.0102

Supplementary Table S4 Metabolic pathways of cecum differential metabolites

Pathway	Ion mode	Diff metabolites	All metabolites	*P*-value
Galactose metabolism	neg	8	25	0.0004
Amino sugar and nucleotide sugar metabolism	neg	10	39	0.0005
Pentose and glucuronate interconversions	neg	10	40	0.0006
Fructose and mannose metabolism	neg	7	28	0.004
Polyketide sugar unit biosynthesis	neg	5	17	0.008
Galactose metabolism	pos	14	24	1.47e-09
Starch and sucrose metabolism	pos	8	9	3.57e-08
Linoleic acid metabolism	pos	13	27	1.29e-07
Carbohydrate digestion and absorption	pos	6	9	3.68e-05
ABC transporters	pos	16	60	4.93e-05

Supplementary Table S5 Metabolic pathways of colonic differential metabolites

Pathway	Ion mode	Diff metabolites	All metabolites	*P*-value
Metabolism of xenobiotics by cytochrome P450	neg	8	25	0.0004
Porphyrin and chlorophyll metabolism	neg	10	39	0.0005
2-Oxocarboxylic acid metabolism	neg	10	40	0.0006
Prolactin signaling pathway	pos	7	28	0.004
Lysine degradation	pos	5	17	0.008
Breast cancer	pos	1	1	0.011
Endocrine and other factor-regulated calcium reabsorption	pos	1	2	0.022

Supplementary Table S6 Differentially abundant metabolites in cecum (Top 10, mean±SD)

Metabolites	Mean mstn	Mean wt	*P*-value
NIACINAMIDE	1137.65±517.18	3052.3±472.13	0.000715
7alpha-hydroxy-3-oxochol-4-en-24-oic Acid	21114.41±14122.61	74162.22±17385.43	0.001451
Acylcarnitine 9:0	16152.04±3317.49	7071.46±1687.41	0.001676
Riboflavin	10184.65±1802.46	4514.19±2417.68	0.004886
3-Oxo-4,6-choladienoic acid	28130.23±18315.29	97946.14±34423.66	0.00564
Ethyl 4-phenylbutanoate	17489.25±4475.08	36935.07±10158.98	0.006014
(2E,6E)-1-Hydroxy-2,6,10-farnesatrien-9-one	6297.5±1930.53	13055.74±3373.98	0.006666
Deoxyadenosine	28471.63±13302.95	60696.67±12253.36	0.007316
CYTIDINE-3'-MONOPHOSPHATE	707.53±141.71	1796.37±645.06	0.007401
Glyceraldehyde	9558.16±6790.4	24010.02±5690.17	0.011492

Supplementary Table S7 Differentially abundant metabolites in colon (Top 10, mean±SD)

Metabolites	Mean mstn	Mean wt	*P*-value
Dihydromaleimide beta-D-glucoside	36069.41±9657.05	5872.23±3078.65	0.000573
L-Dopa	1903.95±454.09	589.29±194.19	0.00106
L-cis-Cyclo(aspartylphenylalanyl)	315.37±99.28	705.29±145.26	0.00197
Methionyl-Tryptophan	967.21±328.03	2134.27±517.1	0.004317
1-(6Z,9Z,12Z-octadecatrienoyl)-glycero-3-phosphate	8067.68±3394.8	28561.98±10622.69	0.004451
Sandosapogenol	8518.55±3915.84	18536.52±3431.83	0.005068
Biocytin	142.74±34.76	372.25±125.69	0.005456
(Z)-3-(1-Formyl-1-propenyl)pentanedioic acid	1439.25±1044.61	7088.11±3016.01	0.005462
Linoleic acid	28503.2±12140.02	59699.95±11503.04	0.005768
FAPy-adenine	1743.79±487.14	3831.46±1143.16	0.007347

Supplementary Table S8 Comparison of CAZy-associated microorganisms in the MSTN and WT groups of cec (mean±SD)

CAZyLevel1	Mean mstn	Mean wt	log2FC	*P*-value

Carbohydrate Esterases	3080.71±194.69	2686.76±163.70	0.20	0.0159
Glycoside Hydrolases	38486.83±1922.22	35606.05±972.24	0.11	0.0635
Glycosyl Transferases	37775.97±732.15	36309.47±2125.62	0.06	0.2857
Carbohydrate-Binding Modules	22467.10±682.22	21021.82±1692.176	0.10	0.2857
Polysaccharide Lyases	2706.50±391.06	2236.69±612.68	0.28	0.2857
Auxiliary Activities	965.83±213.33	782.81±158.75	0.30	0.5556

Supplementary Table S9 Comparison of CAZy-associated microorganisms in the MSTN and WT groups of col (mean±SD)

CAZyLevel1	Mean mstn	Mean wt	log2FC	*P*-value
Carbohydrate Esterases	3053.38±258.94	2830.89±169.87	0.11	0.0405
Auxiliary Activities	662.59±121.36	803.13±208.15	-0.28	0.4127
Glycoside Hydrolases	36914.06±637.69	36600.83±950.99	0.01	0.7302
Carbohydrate-Binding Modules	20076.89±1111.67	20173.50±1067.63	-0.01	0.7302
Polysaccharide Lyases	1846.13±339.51	2062.84±230.99	-0.16	0.7302
Glycosyl Transferases	36971.49±1336.07	36932.69±813.12	0	0.9048
